# A randomized controlled trial of the effect of a nature-based intervention on climate capability and eco-anxiety in teenagers

**DOI:** 10.3389/fpsyg.2025.1648880

**Published:** 2025-09-22

**Authors:** Róise Glynn, Clodagh O. Dwyer, Monica Casey, Santosh Sharma, Pat Harrold, Pat O’Connor, Patrick Harrold, Liam G. Glynn

**Affiliations:** ^1^Ursuline Secondary School, Thurles, Ireland; ^2^School of Medicine and Health Research Institute, University of Limerick, Limerick, Ireland; ^3^Castleconnell River Association, Limerick, Ireland; ^4^Sea to Source Committee, Ballycroy, Ireland

**Keywords:** climate change, randomized controlled trial, adolescents, climate capability, eco-anxiety

## Abstract

**Introduction:**

We are experiencing a climate and biodiversity crisis unprecedented in the history of mankind. Climate Capability is the degree to which individuals have the skills, understanding, and motivation to make behavioral changes that will reduce their individual contribution to climate change; and appreciate the need for collective action and governance to limit the magnitude of climate change and mitigate its effects. This novel randomized controlled trial tested an intervention to change climate capability in teenagers.

**Methods:**

The study design was a randomized controlled trial recruiting teenagers from a single school community in Ireland. At baseline, prior to allocation, climate capability was measured using the climate capability scale in all participants. The intervention consisted of weekly online climate education and motivation messages and a supervised field trip. The primary outcome was change in climate capability score between baseline and follow-up.

**Results:**

A total of 116 students were invited to participate and 86 (73%) agreed to do so and completed baseline data (Intervention = 43; Control = 43); 83 provided outcome data (Intervention = 43; Control = 40). There was evidence of a significant intervention effect (*p* < 0.01); with an increase in mean climate capability score of 8.2 (4.9–11.5) and an associated increase in eco-anxiety score of 7.2 (3.7–10.7) favoring the intervention. There was a statistically significant correlation between change in climate capability and change in eco-anxiety (Pearson correlation = 0.485, *p* < 0.001).

**Discussion:**

The study results appear to confirm the effectiveness of a nature-based intervention in teenagers to build positive relationships with the environment and build their own capacity and capability to effect positive environmental change. Those responsible for developing school curricula should consider including climate education and engagement in the education of young people if we are to ensure that we have enough climate capable citizens of the future. The trial was registered with ISRCTN on 26/10/24 (No: 46298/www.isrctn.com/ISRCTN18655072).

## Introduction

We are experiencing a climate and biodiversity crisis unprecedented in the history of mankind (Almond et al., 2020; IPCC, 2021). Responding effectively requires more than just technological innovation but will also depend on developing “climate capability.” Climate capability is the degree to which individuals have the skills, understanding, and motivation to make behavioral changes that will reduce their individual contribution to climate change; and appreciate the need for collective action and governance to limit the magnitude of climate change and mitigate its effects (Horry et al., 2023). Achieving meaningful change requires citizens who are informed, motivated, and empowered to make behavioral changes and to push for systemic transformation (Prosser and Whitmarsh, 2022).

A linked concept is “eco-anxiety,” or climate anxiety as it is also known, which is described as both a personal and collective emotional response, influenced by perceived threats to the environment, uncertain futures, and inadequate systemic responses (Clayton and Karazsia, 2020). Research on eco-anxiety highlights its prevalence and effects, particularly among young people who want to address climate change but often lack the resources or knowledge to act effectively. Research indicates that the emotional burden associated with climate change can lead to increased anxiety and a sense of disempowerment among young individuals, exacerbating the challenges they face during a critical developmental period (Hickman et al., 2021).

Young people are a particularly important demographic in this context. Frequently described as the leaders of tomorrow, they are both entrusted with and burdened by the responsibility of addressing climate and biodiversity challenges and are thus especially vulnerable to the psychological impacts of climate change. However, it has also been shown that eco-anxiety is positively related to pro-environmental behavior and environmental activism (Ogunbode et al., 2022) and nature-based interventions have garnered attention as promising strategies to mitigate mental health issues related to climate change. Enhancing climate capability among young people could therefore not only reduce their sense of disempowerment but also harness eco-anxiety as a driver of action.

Nature-based interventions typically involve exposure to natural environments and activities designed to promote ecological awareness and foster a deeper connection to nature (Bratman et al., 2019). It has long been established that such experiences can enhance emotional well-being, improve coping mechanisms, and cultivate a sense of agency in individuals confronted with environmental challenges (Kaplan and Kaplan, 1989). Moreover, the restorative effects of nature exposure have been linked to reductions in stress and anxiety levels, highlighting its potential as a therapeutic tool for addressing eco-anxiety specifically (Barton and Pretty, 2010). It has also been shown that participatory learning and experiential education significantly increased environmental awareness and action (Schultz and Zelezny, 1999). More recent studies seem to support this, suggesting that climate education interventions combining knowledge dissemination with community-based activities might be effective in increasing engagement and reducing anxiety (Lawson et al., 2019). The role of nature in fostering resilience and emotional stability has been further supported by studies indicating that individuals who engage with natural settings report higher levels of life satisfaction and lower levels of psychological distress (Mayer et al., 2009). Together, these findings point to the potential of integrating nature-based and educational strategies to strengthen youth resilience and climate engagement, especially for young people struggling with the implications of climate change.

This study is underpinned by “Social Cognitive Theory” (Bandura, 1986), which emphasizes the reciprocal interaction between personal factors, environmental influences, and behavior. Developing climate capability in individuals can be understood through the lens of self-efficacy which is the belief in one’s ability to act effectively. Eco-anxiety, while potentially overwhelming, may enhance motivation when paired with opportunities to learn, practice, and observe constructive action around climate change. Nature-based interventions in educational contexts provide such opportunities, combining experiential learning with supportive environments that foster coping skills, resilience, and agency. In this way, these interventions can transform eco-anxiety from a source of disempowerment into a catalyst for climate action and a sense of climate capability, aligning with the “reciprocal determinism” at the heart of social cognitive theory (Bandura, 1986).

To date, no randomised controlled trials have examined the effect of nature-based interventions on climate capability or eco-anxiety among teenagers as part of educational curricula. It has been suggested that ecological coping mechanisms should be introduced into educational curricula to empower students with knowledge and actionable steps (Pihkala, 2020) but such a suggestion requires a robust evidence base. The aim of this study provides the opportunity to contribute to this evidence base as it explicitly sets out to explore the effect of a nature-based intervention on climate capability and eco-anxiety among teenagers.

## Materials and methods

### Recruitment and intervention

All Transition Year (TY) students (15–16 year old teenagers) in the Ursuline Secondary School, an all-female school, in Thurles, Co Tipperary, Ireland were invited to take part in the study using a recruitment campaign that consisted of posters in the school, intercom and social media announcements and word of mouth. There were no exclusion criteria applied. Through this campaign, all TY students were invited to attend a preliminary meeting at the school with the investigators. At this meeting, all students received a presentation on the climate and biodiversity crisis and on the planned study. After giving them time to consider this information at this meeting, if they wished to participate, students then had to sign an assent form. They were then given a hard copy of the study consent which their parents had to fill out and sign within 1 week if they wished to participate in the study.

Once parental informed consent was received, all participants were assigned a numerical code numbered between 001 and 100 so that all data would be anonymous. Before they were assigned to control or intervention groups, all participants were asked to fill out an online survey link via Qualtrics XM which was emailed to participants. This survey consisted solely of the following two validated measurements tools: the “Climate Capability Scale” (CCS) (Horry et al., 2023) and the “Hogg Eco-anxiety Scale” (HEAS) (Hogg et al., 2021). The Climate Capability Scale is 24-item questionnaire which has been specifically validated for use with adolescents. It measures an individual’s understanding of climate change and its effects; their motivation and willingness to reduce their individual contributions to climate change; and their appreciation of the role of governance and systemic change in tackling climate change (Horry et al., 2023). The Hogg Eco-anxiety Scale is a 13-item scale that captures four dimensions of eco-anxiety: affective symptoms, rumination, behavioural symptoms, and anxiety about one’s negative impact on the planet (Hogg et al., 2021).

Once participants had completed the survey, they were assigned randomly to either intervention or control groups. Randomization occurred using random permuted blocks, to ensure similar numbers of participants in the intervention and control groups. An independent investigator was responsible for generating the allocation sequence using a computer software program^1^ (The National Cancer Institute's Division of Cancer Prevention, 2024). The same independent investigator was responsible for assigning participants to the intervention and control groups after being called at a central site.

The intervention group over the following 4 weeks received weekly GenAI-generated online messages consisting of climate education and motivation to climate action (Appendix A). At the end of the 4 weeks the intervention group undertook a supervised half-day field trip in Castleconnell, Co Limerick on the river Shannon. This trip consisted of climate education, Shinrin Yoku forest bathing, river restoration and tree planting. The control group received their usual educational curriculum during the study period. At the end of 4 weeks follow-up data was then collected on all participants (intervention and control) using the same online survey tool. Enrollment into the study commenced on 6th November, 2024 and all final outcome assessments were completed by the 16th December 2024. The trial was registered with ISRCTN on 29th October, 2024.

### Outcomes

The primary outcome was change in climate capability measured using the climate capability scale (Horry et al., 2023), and the secondary outcome was change in eco-anxiety measured using the Hogg eco-anxiety scale (Hogg et al., 2021) between baseline and follow-up.

### Ethical approval

Ethical approval was granted on 4/11/24 from the EHS Ethics committee at University of Limerick (No: 2024_10_08_EHS).

### Generative artificial intelligence (GenAI)

GenAI was used to generate the weekly climate education and action messages sent to intervention group participants (Appendix A). GenAI had no other role in the study or in the preparation of this manuscript.

### Sample size and statistical analysis

Sample size calculation was based on previous pilot work of the climate capability scale and included the following assumptions: (a) estimated mean climate capability score of 33.54 (Çimşir et al., 2024) (b) 10% change in the control group (c) standard deviation of mean change of climate capability score of 8.81 (d) ability to detect at least 20% relative decrease in climate capability score between intervention and control groups with 80% power at the 0.05 significance level (e) drop-out rate of 10%. Based on these considerations, we estimated that we would require approximately 45 participants per group. The data from the surveys was collected using an Excel database and then this was opened in SPSS to facilitate statistical analyses. Descriptive statistics were used for quantitative variables in calculating mean and standard deviation. For the primary and secondary outcomes, we compared the mean change in climate capability score and eco-anxiety score at baseline and follow-up between intervention and control groups. The mean difference between control and intervention groups was compared using independent samples t-test and Pearson correlation was used to examine the relationship between change in climate capability and change in eco-anxiety in participants between baseline and follow-up. A regression line was fitted to examine the association between changes in climate capability and changes in eco-anxiety among participants from baseline to follow-up. A 5% level of significance was applied to all statistical tests. All statistical analyses were performed using the software package SPSS version 28.01.0.

### Qualitative data collection and analysis

All participants (intervention and control) were invited to take part in focus group interviews regarding their experience of the study and of those who agreed, a purposive sample which included intervention and control participants were selected for interview. The topic guide for the qualitative interviews is described in Appendix B. An iterative approach was taken in order to be responsive to, and incorporate, findings from the data as they emerged (Ziebland and McPherson, 2006). The interviews continued until it was felt that data saturation was reached, at the point where new data collection did not shed any further light in this issue investigation (Glaser and Strauss, 1967) and no new themes emerged. The interview questions were developed by reviewing other qualitative research exploring climate capability and eco-anxiety. These were then discussed within the research team to decide what questions would most thoroughly explore the participants’ experiences. The participants were consented for interview, audio recording and use of anonymous quotations. The final topic guide is described in Appendix B. To enhance reliability, all interviews were recorded on the Microsoft Teams platform and automatically transcribed. Transcriptions were checked for accuracy by listening back to the full audio recording. The five stages of the Framework Process were followed in the examination of the qualitative data which included familiarization, thematic framework identification, indexing, charting, mapping and interpretation (Ritchie and Spencer, 1994). To heighten reflexivity, four members of the research team, (a nurse, a general practitioner and two teenage co-investigators) reviewed all the data and contributed to the categorization, coding and thematic analysis (Richards, 2005).

## Results

### Recruitment and baseline characteristics of trial participants

**Figure 1:**
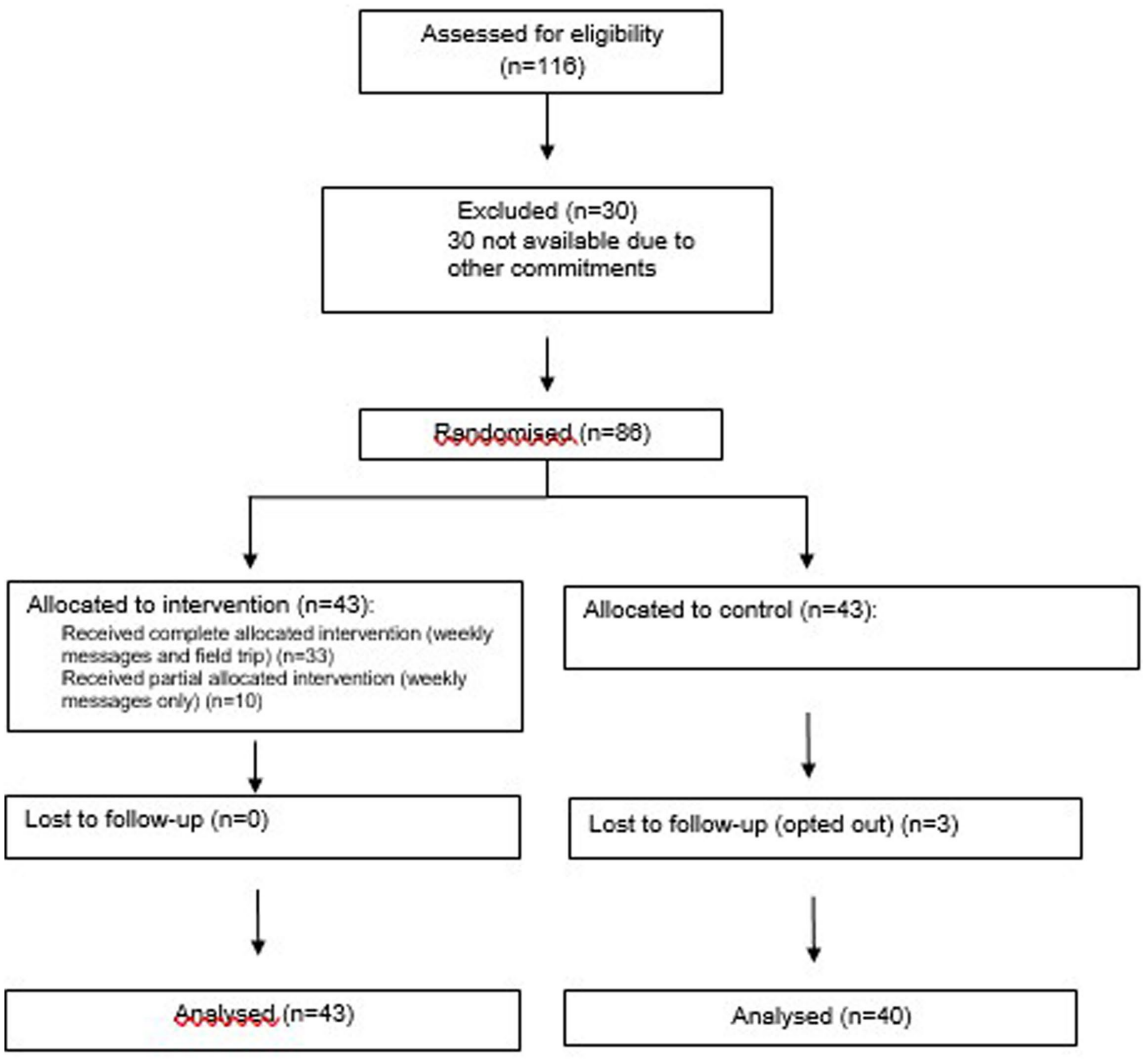
Participant flow through recruitment and follow up.

A total of 116 TY students were invited to participate. Of these 30 (26%) were excluded, due to not being available due to other commitments (Figure 1). Therefore, 86 were eligible and randomized, all were female (as the study site was an all-female school) and aged either 15 or 16 years of age. There were no significant differences between control and intervention groups at baseline for climate capability scores or eco-anxiety scores. The flow chart in Figure 1 represents the movement of participants through the stages of the trial. Of the 86 participants randomized, 83 (97%) completed follow-up. In the intervention group, 33 (77%) received the complete allocated intervention (weekly messages and field trip) while 10 (23%) received partial allocated intervention (weekly messages only) (*n* = 10).

### Outcome data

**Figure 2:**
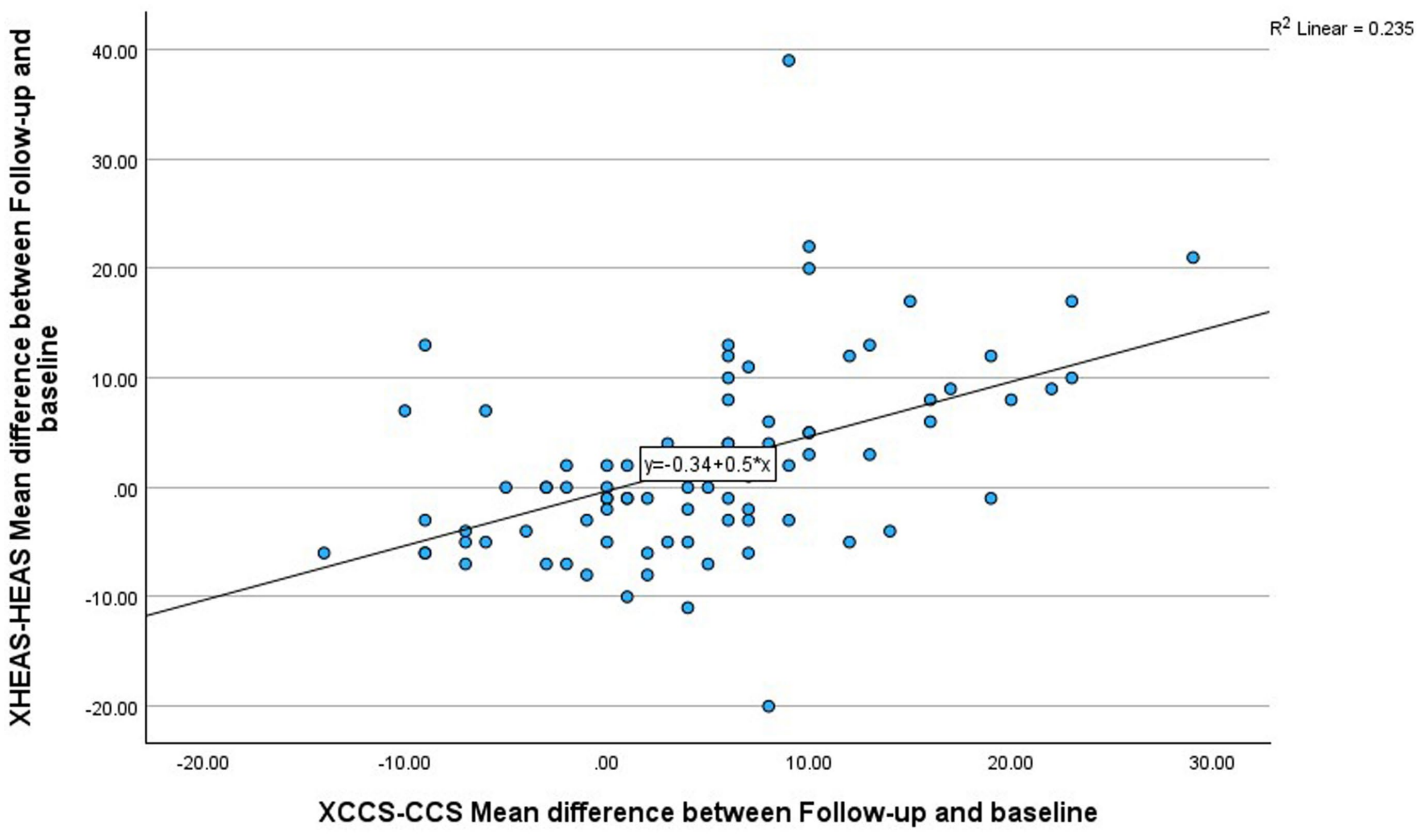
Correlation between change in climate capability score (CCS) and change in eco-anxiety score (HEAS) for all trial participants.

The mean scores and sub-scores at baseline and follow up for control and intervention groups are shown in Table 1 below. In addition, the mean difference, standard deviations and *p* values between baseline and follow up for control and intervention groups are also shown in Table 1. Mean climate capability score at baseline for intervention and control groups was 24.4 (7.3) and 27.7 (8.0), respectively and mean eco-anxiety score at baseline for intervention and control groups was 7.7 (6.5) and 6.7 (5.1), respectively. There was evidence of a significant intervention effect (*p* < 0.01); with an increase in mean climate capability score of 8.2 (95% Confidence Interval 4.9–11.5) and an increase in eco-anxiety score of 7.2 (5% Confidence Interval 3.7–10.7) favoring the intervention. In addition, there was a statistically significant correlation between change in climate capability and change in eco-anxiety (Pearson correlation 0.485, *p* < 0.01) as shown in Figure 2. In regard to the eco-anxiety sub-scores, there was evidence of a significant intervention effect (*p* < 0.01) with an increase in eco-anxiety *affective* subscore of 1.9 (95% Confidence Interval 0.7–3.1) favoring the intervention; an increase in eco-anxiety *rumination* subscore of 2.4 (95% Confidence Interval 1.5–3.4) favoring the intervention; and an increase in eco-anxiety *personal impact* subscore of 2.2 (95% Confidence Interval 1.1–3.4) favoring the intervention; with no statistically significant change in eco-anxiety *behavioral* subscore.

**Table 1:** Mean baseline and follow-up and mean difference for primary outcomes in control and intervention groups.

Primary Outcomes	*n*	Baseline Mean (SD)	*n*	Follow-up Mean (SD)	Mean difference (SD)	*p*
Eco-anxiety (EA) Score						
Control Group	43	6.67 (5.07)	40	5.00 (5.95)	−1.73 (5.07)	*p* < 0.001
Intervention Group	43	7.74 (6.49)	43	13.36 (9.25)	+5.51 (10.14)	
EA Affective Sub-score						
Control Group	43	1.98 (1.95)	40	1.45 (2.25)	−0.53 (1.68)	*p* = 0.001
Intervention Group	43	2.40 (2.16)	43	3.74 (2.99)	+1.35 (3.43)	
EA Rumination Sub-score						
Control Group	43	2.05 (1.70)	40	1.15 (1.63)	−0.90 (1.58)	*p* < 0.001
Intervention Group	43	2.07 (2.10)	43	3.61 (2.41)	+1.54 (2.62)	
EA Behavioral Sub-score						
Control Group	43	0.21 (0.51)	40	0.60 (1.36)	+0.38 (1.41)	*p* = 0.081
Intervention Group	43	0.93 (1.72)	43	2.00 (2.44)	+1.07 (2.81)	
EA Personal Impact Sub-score						
Control Group	43	2.44 (2.02)	40	1.80 (2.12)	−0.68 (2.19)	*p* < 0.001
Intervention Group	43	2.35 (2.15)	43	3.91 (2.52)	+1.56 (3.07)	
Climate Capability Score						
Control Group	43	27.70 (7.99)	40	28.30 (7.78)	+0.50 (6.36)	*p* < 0.001
Intervention Group	43	28.42 (7.32)	43	37.09 (8.56)	+8.67 (8.56)	

### Qualitative data

All participants were invited to interview (n = 86) and eight (9%) agreed to be interviewed, four from the control group and four from the intervention group. Interviews were semi-structured and were conducted by two members of the research team (RG and CD) for quality control purposes. Respondents were assigned a numerical code as follows: CR1 was the first respondent from the control group; IR1 was the first respondent from the intervention group and so on. After following the stages of framework analysis, the themes that emerged from the data were classified into three major interrelated themes: powerlessness; responsibility; and capability and hope.

### Powerlessness

There was a general sense of powerlessness in the face of the very big problem that is climate change and the ecological crisis.:

“I feel like it's doing irreparable damage, and I don't have the power to fix it by myself.” CR4

There was also a notable lack of agency which is understandbale in the context of the age of the study participants.

“Its just that we can’t really do anything ….by ourselves.” IR2

### Responsibility

On the one hand there was a frustration that this problem now seemed to be the responsibility of the younger generation even though it was not caused by them:

“They have completely just destroyed the world and now it's up to us to fix it.” IR1

However, the intervention group did seem to develop an awareness as a result of the study of what they could do to make a difference and the importance of doing something:

“I definitely worry for future generations because I want them to know what a polar bear is, and I want them to know all the beautiful things that we've grown up knowing.” IR1

### Capability and hope

The most striking theme in the data emerged from the intervention group which was the a renewed sense of hope as a result of the intervention experience:

“There is definitely a chance that we can fix the world and make it what it was.” IR3

It was also evident that the intervention provided a new sense of self-effiacy and capability even in the face of such a large and complex problem as climate change:

“This made me feel better like I am doing my part to save the world” IR2

## Discussion

### Summary of main findings

In this novel trial of a nature climate education and action intervention in TY students, there was evidence of a significant intervention effect (*p* < 0.01) with an increase in mean climate capability score and an associated increase in eco-anxiety score favoring the intervention over the four-week period of the trial. Eco-anxiety did not decrease with increased climate capability, but instead increased alongside it, suggesting a more complex and potentially adaptive relationship between eco-anxiety and climate capability in this age group. This change was also seen in the qualitative data where the intervention appeared to engender a sense of climate capability with an associated increase in agency and optimism to counteract the powerlessness felt at the beginning of the study.

### Comparison with existing literature

Our findings align with previous research suggesting that eco-anxiety can be both a burden and a motivator (Clayton and Karazsia, 2020; Hickman et al., 2021). In addition, it has been shown that climate distress positively predicts pro-environmental behavior, supporting the view that eco-anxiety, when coupled with coping strategies, can serve as an adaptive emotional response (Ogunbode et al., 2022). Similarly, the importance of the role of emotional arousal has been highlighted in bridging the intention–behavior gap in environmental action (Bamberg et al., 2015).

A reliable and valid measure of climate capability has many applications, including tracking population-level changes in climate capability over time and evaluating the effectiveness of educational programmes, public engagement activities, and public messaging campaigns (Horry et al., 2023). This study is first to use the climate capability scale as the primary outcome in a randomized controlled trial of a nature-based intervention in teenagers. Adolescents tend to have lower climate capability scores than adults and this was confirmed in our study where the scores seen were lower than those measured previously in adult populations (Horry et al., 2023). Climate scepticism was low in this age group with only 7% of participants indicating that they believed that scientists are exaggerating the effects of climate change. These results are broadly in line with previous research which has documented that climate change scepticism is a minority view generally in Western Europe (Poortinga et al., 2011).

The majority of young people appear to believe that climate change is a significant threat to their safety (Australia U, 2019). With the apparent universality of this experience for young people, it is important to consider if this eco-anxiety can be harnessed in a positive way and mitigated in terms of its effects on mental health. It would appear from our study, that this anxiety in teenagers does not lead to eco-paralysis but rather has the potential to trigger a motivation to action and an increase in climate capability. While seemingly counterintuitive, this suggests that concern about one’s personal impact on the planet, a form of eco-anxiety, does not necessarily lead to negative mental health and might even encourage pro-environmental actions. Eco-anxiety is this age group can therefore be seen as a healthy response to a challenging reality and a motivator for positive change. The relationship between eco-anxiety, climate capability and positive environmental behavior is important, especially because engaging in positive environmental behavior may prove to be an effective strategy for coping with eco-anxiety (Bamberg et al., 2015). In addition, different levels of eco-anxiety can have different effects on pro-environmental behavior. It has been hypothesized that moderate levels of climate anxiety may be optimal for pro-environmental behavioral response (Coates et al., 2024); however in the age group under study (15 to 16 year old females) this did not seem to be the case with high levels of climate capability seen even with higher eco-anxiety scores.

Social cognitive theory suggests that behavior is shaped by reciprocal interactions between personal factors (cognitions, emotions, self-efficacy), environmental influences (social norms, learning contexts), and behavior itself with the concept of self-efficacy of central importance (Bandura, 1986). Climate capability aligns closely with this construct as does the potential of the intervention under study to influence climate capability. As the study participants gained knowledge and participated in pro-environmental action (tree planting, river restoration), they developed stronger beliefs in their capacity to contribute meaningfully to climate solutions. This was evident in the qualitative data where accounts of feeling more capable and hopeful were evident. Consistent with social cognitive theory, vicarious learning and experiential mastery were key (Bandura, 1986) as study participants observed peers engaging in climate action, directly practiced such behaviors, and received social reinforcement in a supportive school environment.

A key question is whether the observed increases in climate capability and eco-anxiety will persist over time. Social cognitive theory would predict that sustained self-efficacy and reinforcement are required to maintain pro-environmental action. It is likely that without continued opportunities for mastery and social support, anxiety could revert to paralysis. Future interventions should therefore embed ongoing participatory opportunities within educational curricula.

### Strengths and limitations

The strengths of this study are the randomized design, allocation concealment, the high completion rate for participants (97%) and the limited number of exclusion criteria. The relatively small number of potential participants that did not participate should strengthen the external validity of the results (Rothwell, 2005) and may help to facilitate implementation particularly with such an open recruitment strategy. However, this study also had number of limitations. It was a small study conducted in a single school year among females only and had a short follow up. In addition, some of the intervention group (23%) did not receive the full intervention.

## Conclusion

This study has demonstrated that a nature-based intervention in teenagers can build positive relationships with the environment and build their own capacity and capability to effect positive environmental change. Those responsible for developing school curricula should consider including climate education and engagement in the education of young people if we are to ensure that we have enough climate capable citizens of the future. This trial represents an important step forward in the challenging issue of understanding young people’s knowledge of, and motivation to, become involved in climate action. This study uniquely contributes to climate capability and eco-anxiety research, and further research is required to elucidate clearly the relationship between climate capability and eco-anxiety in this and other age groups.

### Implications for research and practice

It has been suggested that significant improvements in public health in the future are more likely to come from behavioral change rather than from technological or scientific innovation (Schroeder, 2007). The results of the current study would suggest that interventions which reconnect and engage young people with the environment and the issues around climate change may trigger significant increases in climate capability. A correlated increase in eco-anxiety levels is seen but this does not appear to lead to eco-paralysis.

However, structural and developmental constraints need to be considered as teenagers may feel capable and motivated yet lack avenues for significant behavioral impact, leading to frustration. Interventions should therefore expand the focus from individual action to collective efficacy and systemic advocacy, empowering young people to see themselves as part of wider social movements.

Those responsible for developing school curricula should consider including climate education and engagement in the education of young people if we are to ensure that we have enough climate capable citizens of the future. The type of information and learning experience provided can have a profound effect on the outcomes of successful learning and behavior change. This is especially true for climate change information where personal relevance to the issue, or an engaged learning experience, can be critical to solidifying a lasting change, especially for children and young people (Monroe et al., 2019).

There was no statistically significant change in eco-anxiety behavioral subscore in this study which is probably explained by the fact that teenagers do not have the same autonomy as adults with choice and decision-making regarding finances, transport or living arrangements which are generally in the hands of their responsible adults or guardians. Social cognitive theory emphasizes that behavior is constrained not only by motivation but also by opportunity structures and many of the high-impact pro-environmental decisions remain under parental control for this age group. This suggests that while interventions can boost self-efficacy and emotional engagement, behavioral enactment may require broader structural and familial alignment.

The concept of “outcome expectancy,” described as part of social cognitive theory as the results one anticipates from having performed a task, is also relevant to the study results. Participants in the intervention group developed stronger beliefs that collective and systemic responses are possible and necessary, even if their individual contributions were modest. This aligns with prior work showing that participatory, experiential climate education strengthens both efficacy and belief in collective solutions (Lawson et al., 2019; Monroe et al., 2019).

An important direction for future research will be identifying where adolescents perceive that they have the agency and opportunity to make pro-environmental changes to their own behaviors, and where they perceive opportunities to influence the behaviors of their families, peers, and local communities (Horry et al., 2023).

## Data Availability

The datasets presented in this study can be found in online repositories. The names of the repository/repositories and accession number(s) can be found in the article/Supplementary material.

## Footnotes

^1^ https://ctrandomization.cancer.gov/

## References

[ref1] Almond R. E. A. Grooten M. Petersen T. (2020). Living Planet Report 2020: Bending the curve of biodiversity loss

[ref2] Australia U. (2019) A climate for change: 2019 Young Ambassador report

[ref3] Bamberg S. Rees J. Seebauer S. (2015). Collective climate action: determinants of participation intention in community-based pro-environmental initiatives. J. Environ. Psychol. 43, 155–165. doi: 10.1016/j.jenvp.2015.06.006

[ref4] Bandura A. (1986). Social foundations of thought and theory: A social cognitive theory.

[ref5] Barton J. Pretty J. (2010). What is the best dose of nature and green exercise for improving mental health? A multi-study analysis. Environ. Sci. Technol. 44, 3947–3955. doi: 10.1021/es903183r, PMID: 20337470

[ref6] Bratman G. N. Anderson C. B. Berman M. G. Cochran B. de Vries S. Flanders J.. (2019). Nature and mental health: an ecosystem service perspective. Sci. Adv. 5:eaax0903. doi: 10.1126/sciadv.aax0903, PMID: 31355340 PMC6656547

[ref7] Change., I. P. o. C. (2021). The Physical Science Basis. Contribution of Working Group I to the Sixth Assessment Report of the Intergovernmental Panel on Climate Change.

[ref8] Çimşir E. Şahin M. D. Akdoğan R. (2024). Unveiling the relationships between eco-anxiety, psychological symptoms and anthropocentric narcissism: the psychometric properties of the Turkish version of the Hogg eco-anxiety scale. Cambridge Prisms 11:e26. doi: 10.1017/gmh.2024.20, PMID: 38572253 PMC10988136

[ref9] Clayton S. Karazsia B. T. (2020). Development and validation of a measure of climate change anxiety. J. Environ. Psychol. 69:101434. doi: 10.1016/j.jenvp.2020.101434

[ref10] Coates Z. Kelly M. Brown S. (2024). The relationship between climate anxiety and pro-environment behaviours. Sustainability 16:5211. doi: 10.3390/su16125211

[ref11] Glaser B. Strauss A. (1967). The discovery of grounded theory: Strategies for qualitative research: Aldine Publishing Company.

[ref12] Hickman C. Marks E. Pihkala P. Clayton S. Lewandowski R. E. Mayall E. E.. (2021). Climate anxiety in children and young people and their beliefs about government responses to climate change: a global survey. Lancet Planet Health 5, e863–e873. doi: 10.1016/s2542-5196(21)00278-3, PMID: 34895496

[ref13] Hogg T. L. Stanley S. K. O'Brien L. V. Wilson M. S. Watsford C. R. (2021). The hogg eco-anxiety scale: development and validation of a multidimensional scale. Glob. Environ. Chang. 71:102391. doi: 10.1016/j.gloenvcha.2021.102391

[ref14] Horry R. Rudd J. A. Ross H. Skains R. L. (2023). Development and validation of the climate capability scale. Sustainability 15:11933. doi: 10.3390/su151511933

[ref15] IPCC (2021). Climate mate Change e 2021: The Physical Science Basis. Contribution of Working Group I to the Sixth Assessment Report of the Intergovernmental Panel on Climate Change

[ref16] Kaplan R. Kaplan S. (1989). The experience of nature: A psychological perspective: Cambridge University Press.

[ref17] Lawson D. Stevenson K. Carrier S. Strnad R. Seekamp E. (2019). Children can foster climate change concern among their parents. Nat. Clim. Chang. 9, 1–5. doi: 10.1038/s41558-019-0463-3

[ref18] Mayer F. Frantz C. Bruehlman-Senecal E. Dolliver K. (2009). Why is nature beneficial? The role of connectedness to nature. Environ. Behav. 41, 607–643. doi: 10.1177/0013916508319745

[ref19] Monroe M. C. Plate R. R. Annie O. Alison B. Chaves W. A. (2019). Identifying effective climate change education strategies: a systematic review of the research. Environ. Educ. Res. 25, 791–812. doi: 10.1080/13504622.2017.1360842

[ref20] Ogunbode C. A. Doran R. Hanss D. Ojala M. Salmela-Aro K. van den Broek K. L.. (2022). Climate anxiety, wellbeing and pro-environmental action: correlates of negative emotional responses to climate change in 32 countries. J. Environ. Psychol. 84:101887. doi: 10.1016/j.jenvp.2022.101887

[ref21] Pihkala P. (2020). Eco-anxiety and environmental education. Sustainability 12:10149. doi: 10.3390/su122310149

[ref22] Poortinga W. Spence A. Whitmarsh L. Capstick S. Pidgeon N. F. (2011). Uncertain climate: an investigation into public scepticism about anthropogenic climate change. Glob. Environ. Chang. 21, 1015–1024. doi: 10.1016/j.gloenvcha.2011.03.001

[ref23] Prosser A. Whitmarsh L. (2022). Net zero: A review of public attitudes & behaviours.

[ref24] Richards L. (2005). Handling qualitative data a practical guide: Sage Publications.

[ref25] Ritchie J. Spencer L. (1994). “Qualitative data analysis for applied policy research” in Analyzing Qualitative Data. ed. Bryman A. B. (Routledge).

[ref26] Rothwell P. M. (2005). External validity of randomised controlled trials: "to whom do the results of this trial apply?". Lancet 365, 82–93. doi: 10.1016/s0140-6736(04)17670-8, PMID: 15639683

[ref27] Schroeder S. A. (2007). Shattuck lecture. We can do better--improving the health of the American people. N. Engl. J. Med. 357, 1221–1228. doi: 10.1056/NEJMsa073350, PMID: 17881753

[ref28] Schultz W. Zelezny L. (1999). Values as predictors pf environmental attitudes: evidence for consistency across 14 countries. J. Environ. Psychol. 19, 255–265. doi: 10.1006/jevp.1999.0129

[ref29] The National Cancer Institute's Division of Cancer Prevention. (2024) Clinical Trial Randomization Tool

[ref30] Ziebland S. McPherson A. (2006). Making sense of qualitative data analysis: an introduction with illustrations from DIPEx (personal experiences of health and illness). Med. Educ. 40, 405–414. doi: 10.1111/j.1365-2929.2006.02467.x, PMID: 16635119

