# Supplementary material for: A randomized controlled trial of the effect of a nature-based intervention on climate capability and eco-anxiety in teenagers
Source: Front Psychol. 2025 Sep 22;16:1648880. doi: 10.3389/fpsyg.2025.1648880 (PMC12497743; doi:10.3389/fpsyg.2025.1648880)
Supplement: Supplementary file 1 [file Data_Sheet_1.docx]

**Appendix A. Weekly climate education and action messages generated by GenAI (ChatGPT version 4.0)**

**Message 1: Intro to Climate Change 🌍**

Hey there! Have you heard the term “climate change” a lot but aren't sure what it’s about? 🌡️ It’s when greenhouse gases (mostly from burning fossil fuels) trap heat in our atmosphere, causing the Earth to warm up faster than normal. This messes with weather patterns, causes extreme weather, and threatens ecosystems.

**Action:** You can start small! Try turning off the lights when you leave a room, or bike/walk instead of asking for a ride. Little changes in your daily routine add up! 💡🚲

**Message 2: Fast Fashion Facts 👚👖**

Fast fashion (think cheap, trendy clothes) often ends up in landfills fast, too. This industry produces more emissions than all international flights and shipping combined. 😮 Plus, it uses a lot of water and sometimes involves unsafe working conditions.

**Action:** Try thrift shopping, swapping clothes with friends, or repurposing older pieces! Share a look you’ve created without buying new. Let’s make reusing a trend! 💚♻️

**Message 3: Energy and Your Choices 🔌**

The way we use energy matters. Leaving electronics plugged in, even if they’re off, still drains energy and adds to emissions. This is called "vampire power." 👻

**Action:** Unplug chargers and devices when you’re not using them and see if you can get others in your family to join! Look around: which appliances can you unplug right now? ⚡

**Message 4: Trees are Climate Heroes 🌳**

Trees absorb CO₂, helping cool our planet naturally! 🌲 But deforestation (cutting down forests for farming, wood, etc.) means we lose about 10 million hectares of trees each year. That’s like losing 27 soccer fields of trees every minute!

**Action:** Support reforestation efforts by joining a tree-planting day or use search engines like Ecosia that plant trees with every search. You can even start a “green space” in your neighborhood by planting native plants! 🌱

**Appendix B. Topic guide for Qualitative Interviews**

**Introduction**

1. Can you describe how you feel when you think about climate change and its impacts on the planet?
   - (Follow-up: Have these feelings changed over time?)

**Eco-Anxiety**

1. Have you ever experienced feelings of eco-anxiety, such as worry, fear, or helplessness about the environment? If so, how would you describe these feelings?
   - (Follow-up: How frequently do these feelings arise for you?)
2. How do your emotions regarding climate change affect your daily life, if at all?
   - (Follow-up: Do they influence your mental or physical health?)
3. In your opinion, are there specific events or sources of information that tend to trigger or intensify your eco-anxiety?
4. Did the action you took part in as part of this study change how you felt about the environment in any way> If so, how?

**Perceptions of Climate Change**

1. How informed do you feel about the current state of climate change and its impacts?
   - (Follow-up: Where do you get most of your information about climate change?)
2. Do you believe climate change affects you personally? If so, in what ways?
3. Do you discuss climate change with family, friends, or colleagues? If so, how do those conversations usually go?
   - (Follow-up: Do these discussions ease or increase your anxiety?)

**Climate Action Engagement**

1. Have you taken any personal actions to address climate change, such as changes in your lifestyle, participation in activism, or political engagement? If yes, can you describe these actions?
2. What motivates or demotivates you when it comes to engaging in climate action?

- (Follow-up: Do feelings of eco-anxiety influence your level of engagement?)

1. Do you think individual actions such as the action you took part in as part of this study are effective in addressing climate change, or do you feel the responsibility lies more with governments, businesses, and large institutions?
2. Have you faced any barriers (emotional, social, financial, etc.) that make it difficult for you to participate in climate action?

**Emotional Impact of Climate Action**

1. How does participating in climate action such as the action you took part in as part of this study affect your emotional well-being? Does it help alleviate or heighten your eco-anxiety?
2. Looking forward, how do you feel about the future in relation to climate change? Does the prospect of climate action make you more hopeful or more anxious?

**Conclusion**

- Is there anything else you’d like to add about your experience with eco-anxiety and climate action that we haven’t covered?
